# Supplementary material for: A Likelihood Approach for Real-Time Calibration of Stochastic Compartmental Epidemic Models
Source: PLoS Comput Biol. 2017 Jan 17;13(1):e1005257. doi: 10.1371/journal.pcbi.1005257 (PMC5240920; doi:10.1371/journal.pcbi.1005257)
Supplement: S1 File — (TAR.GZ) [file pcbi.1005257.s014.tar.gz › HSPH_Online-SI-Revision/Additions/EnKF08/n100-extreme_noisy/n100-extreme_noisy_EnKF_table0.pdf]

1	R0	{2.76052, 16.3913}	{2.64901, 3.57425}	{{2.24076, 0.283533}, {3., 6.13063}}	{12.5411, 78.1942}	inf	26 580.	5461.34	{4188., 7814.29}	81.3687	0.751527	newly	10 934.9	10 048.1	{3275., 17 836.}	76.3211	0.352901	Reff	0.831027	0.617311	{0.207297, 1.08896}	56.5143	ar	92 090.	189 830.	{174 600., 204 103.}	106.135
2	R0	{2.18202, 15.1734}	{2.59972, 12.2219}	{{2.02724, 3.715}, {3., 19.81}}	{22.1121, 44.515}	inf	17 999.	2621.23	{320.018, 3190.21}	90.4106	1.12103	newly	8027.	1679.66	{202., 3011.}	89.1693	1.00448	Reff	0.873157	0.624544	{0.179025, 1.47514}	68.8255	ar	84 264.	182 717.	{167 799., 210 943.}	116.839
3	R0	{2.57891, 12.5971}	{2.05044, 5.56564}	{{0.287475, 1.54969}, {3., 17.1674}}	{39.7594, 72.5086}	inf	22 711.	8022.32	{455.782, 8039.81}	89.8284	1.0494	newly	11 421.	8650.69	{309., 16 117.}	91.3056	0.768177	Reff	0.727149	0.450231	{0.0523308, 1.04884}	58.2485	ar	90 302.	196 951.	{174 220., 240 726.}	118.102
4	R0	{2.5856, 9.28508}	{2.06408, 6.61404}	{{0.802962, 3.16094}, {3., 9.18437}}	{36.6843, 28.7671}	inf	23 144.	3241.36	{214.185, 2370.36}	91.9349	1.32416	newly	15 345.	2333.62	{159., 1845.}	90.5456	1.26016	Reff	0.760942	0.316093	{0.0952842, 0.648405}	60.4931	ar	90 312.	181 234.	{169 949., 195 130.}	100.675
5	R0	{2.4406, 12.1341}	{2.52569, 5.68553}	{{1.53325, 3.60149}, {3., 11.1427}}	{23.5598, 53.1442}	inf	22 895.	10 804.9	{749.285, 32 694.8}	94.1114	0.865605	newly	12 782.	10 189.9	{661., 41 254.}	98.7968	0.671474	Reff	0.972189	0.600011	{0.10019, 1.13505}	58.9761	ar	88 597.	196 354.	{166 305., 296 617.}	121.626
6	R0	{2.72155, 15.4094}	{2.80321, 5.51297}	{{2.25693, 3.79508}, {3., 6.48792}}	{11.528, 64.2233}	inf	26 147.	5647.47	{2926.64, 12 156.8}	82.2334	0.828774	newly	11 271.	6702.62	{1981., 16 338.}	75.1671	0.534593	Reff	0.880857	0.615265	{0.197092, 0.928183}	43.0172	ar	91 682.	182 178.	{169 027., 197 232.}	98.706
7	R0	{2.63781, 14.3935}	{2.32564, 7.34299}	{{1.18005, 2.45905}, {3., 10.3919}}	{29.9588, 48.984}	inf	24 939.	3179.04	{345.182, 4095.77}	94.576	1.3659	newly	11 237.	2558.99	{191., 3925.}	92.1247	1.13705	Reff	0.790499	0.426698	{0.11397, 0.629682}	52.2552	ar	91 192.	174 573.	{163 138., 182 072.}	91.4348
8	R0	{2.82391, 16.104}	{2.77763, 4.03151}	{{2.24376, 0.473942}, {3., 6.2121}}	{10.1194, 74.9658}	inf	27 368.	5750.58	{1992.73, 8861.93}	82.4586	0.895885	newly	11 207.	8262.45	{2141., 11 950.}	79.7963	0.513265	Reff	0.861801	0.521926	{0.11691, 0.785364}	49.9591	ar	92 664.	189 608.	{173 271., 198 818.}	104.619
9	R0	{2.80645, 14.6457}	{2.76192, 4.20469}	{{2.32258, 0.647172}, {3., 6.29722}}	{10.6182, 71.2906}	inf	27 606.	5009.17	{2904.4, 10 108.}	82.9401	0.902501	newly	12 694.	8431.36	{2328., 13 203.}	75.2439	0.512459	Reff	0.94945	0.623398	{0.371737, 0.867153}	45.3263	ar	92 567.	176 679.	{161 498., 185 403.}	90.8666
10	R0	{1.86213, 5.4271}	{2.2228, 7.59549}	{{0.0586001, 2.14431}, {3., 11.1826}}	{59.7435, 68.7778}	inf	10 896.	6932.63	{2964.18, 9023.57}	62.5586	0.369352	newly	11 765.	6131.74	{2376., 9663.}	69.9334	0.471507	Reff	0.763157	0.600617	{0.0585995, 0.698697}	39.698	ar	75 362.	195 323.	{179 720., 209 637.}	159.179
11	R0	{2.27349, 14.6338}	{2.25786, 6.28505}	{{0.760031, 1.76497}, {3., 10.916}}	{40.462, 57.0511}	inf	19 721.	3276.19	{223.956, 4243.38}	93.8312	1.30061	newly	8893.	2626.21	{184., 4141.}	93.7397	1.0508	Reff	0.854264	0.397147	{0.0414903, 0.666042}	53.51	ar	85 805.	178 892.	{167 494., 190 173.}	108.487
12	R0	{2.77261, 14.1894}	{2.56336, 5.8229}	{{1.86026, 2.9327}, {3., 8.0779}}	{18.0448, 58.963}	inf	27 138.	3474.02	{925.521, 4989.45}	93.0888	1.18463	newly	12 745.	3760.69	{569., 6942.}	91.7528	0.933031	Reff	0.865526	0.395521	{0.0868308, 0.60511}	56.6197	ar	92 508.	180 780.	{168 212., 189 096.}	95.4213
13	R0	{2.65402, 19.3935}	{2.3844, 9.73422}	{{0.0689499, 3.89412}, {3., 13.6027}}	{28.6701, 49.8068}	inf	25 765.	2673.04	{271.57, 6137.66}	89.6253	1.26525	newly	8948.	1991.93	{255., 4309.}	83.9342	1.0216	Reff	0.987375	0.47972	{0.014174, 1.99993}	78.3386	ar	91 184.	182 692.	{170 097., 229 148.}	100.355
14	R0	{1.75297, 4.45354}	{2.49832, 5.36437}	{{1.55667, 3.54757}, {3., 11.4232}}	{48.9077, 45.2768}	inf	10 727.	11 825.6	{2015.48, 24 938.7}	103.672	0.443568	newly	15 310.	13 078.1	{1419., 42 906.}	84.676	0.510151	Reff	0.926059	0.754741	{0.432943, 0.953478}	41.2902	ar	71 263.	204 028.	{173 755., 302 857.}	186.302
15	R0	{1.99928, 7.1501}	{2.32719, 12.8959}	{{1.43396, 3.8859}, {3., 19.2773}}	{36.2874, 106.583}	inf	13 996.	12 400.7	{1915.31, 31 672.2}	94.9024	0.521169	newly	12 056.	8515.71	{1022., 36 231.}	96.0552	0.673789	Reff	0.80563	0.788675	{0.202654, 1.44232}	44.2989	ar	79 602.	211 281.	{176 363., 302 918.}	165.422
16	R0	{2.67398, 10.3134}	{2.48173, 11.1893}	{{1.48384, 4.08246}, {3., 19.8251}}	{19.9678, 50.4997}	inf	23 578.	12 249.6	{1471.8, 42 336.5}	88.1089	0.749226	newly	14 011.	8954.6	{997., 31 116.}	98.4521	0.768743	Reff	0.705449	0.638281	{0.133262, 1.49069}	72.7059	ar	91 289.	206 467.	{171 548., 298 218.}	126.168
17	R0	{2.44634, 16.3226}	{2.64962, 9.70341}	{{2.18516, 3.9335}, {3., 13.8705}}	{17.4911, 40.5523}	inf	22 666.	2573.04	{114.596, 2574.23}	90.4657	1.39033	newly	9419.	2344.71	{59., 5579.}	89.5716	1.19469	Reff	0.907103	0.665999	{0.186999, 1.11471}	42.3009	ar	88 710.	179 625.	{167 686., 204 973.}	102.486
18	R0	{2.02168, 10.1676}	{2.1416, 6.63429}	{{0.203242, 2.63952}, {3., 10.4607}}	{53.1057, 37.0138}	inf	15 535.	2056.63	{122.085, 1873.11}	93.3296	1.31827	newly	10 392.	1649.3	{107., 1848.}	91.2567	1.24959	Reff	0.996001	0.348975	{0.0436, 0.644853}	65.5158	ar	80 299.	170 482.	{163 373., 172 597.}	112.309
19	R0	{2.89969, 15.009}	{2.7815, 4.29069}	{{2.25411, 0.483717}, {3., 6.23119}}	{8.84976, 71.4125}	inf	28 996.	3912.86	{2662.46, 8104.55}	86.5152	0.939017	newly	12 862.	6004.49	{2024., 10 089.}	78.8046	0.597657	Reff	0.881738	0.423674	{0.225101, 0.63117}	59.6085	ar	93 349.	174 969.	{166 607., 196 377.}	87.4355
20	R0	{2.68933, 7.34928}	{2.21336, 5.02722}	{{1.26483, 2.19139}, {3., 7.67437}}	{29.0194, 34.5411}	inf	24 920.	2039.23	{340.11, 3603.32}	91.8169	1.34456	newly	20 039.	4077.58	{271., 9836.}	90.1254	1.24716	Reff	0.765733	0.48422	{0.0301608, 0.753131}	49.6907	ar	91 574.	188 001.	{176 069., 215 861.}	105.3
21	R0	{2.15352, 9.28074}	{2.55571, 7.91737}	{{1.53248, 3.64958}, {3., 19.6955}}	{32.505, 53.3732}	inf	17 876.	9262.13	{1407.87, 15 937.4}	94.4206	0.756922	newly	12 361.	10 024.9	{840., 41 286.}	112.182	0.727768	Reff	0.8829	0.691176	{0.08655, 1.53112}	54.3911	ar	83 845.	209 729.	{183 018., 305 222.}	150.139
22	R0	{2.21953, 9.04387}	{2.50472, 9.55949}	{{1.91472, 4.18228}, {3., 14.8003}}	{24.1102, 44.3693}	inf	18 399.	6375.05	{1547.9, 11 589.3}	75.9213	0.695977	newly	13 126.	6787.	{1103., 13 798.}	86.8635	0.709102	Reff	0.851523	0.850577	{0.444171, 1.9147}	47.0777	ar	84 856.	217 872.	{192 065., 294 488.}	156.755
23	R0	{2.21987, 14.5347}	{2.62419, 10.9598}	{{2.13633, 3.62954}, {3., 17.6336}}	{21.1467, 41.579}	inf	18 816.	3364.58	{257.656, 7595.72}	86.3444	1.08602	newly	8717.	3312.93	{96., 9027.}	80.7745	0.908011	Reff	0.868968	0.804351	{0.3344, 2.07418}	59.659	ar	85 067.	195 669.	{172 510., 259 197.}	130.017
24	R0	{2.84268, 17.9659}	{2.38354, 12.7213}	{{1.6245, 3.38025}, {3., 18.4964}}	{22.2395, 31.7845}	inf	27 580.	4937.55	{552.296, 6842.47}	82.2242	1.0207	newly	10 377.	3506.29	{458., 6112.}	86.5997	0.892053	Reff	0.920602	0.773706	{0.20012, 1.62948}	62.2473	ar	92 836.	203 478.	{174 697., 253 487.}	119.18
25	R0	{2.52706, 10.3679}	{2.49018, 5.66684}	{{1.82801, 2.99476}, {3., 8.17025}}	{20.9229, 45.3424}	inf	22 248.	3041.14	{1551.16, 4184.97}	87.7302	0.972059	newly	13 481.	3595.72	{1178., 4871.}	87.0533	0.83173	Reff	0.761075	0.378998	{0.0193402, 0.585168}	60.448	ar	89 623.	179 063.	{166 072., 212 990.}	99.7962
26	R0	{2.62835, 19.1088}	{2.59815, 9.42338}	{{2.19421, 4.13372}, {3., 14.5344}}	{18.3999, 50.6857}	inf	24 689.	2059.11	{190.203, 10 072.8}	91.6598	1.47581	newly	8698.	2196.27	{131., 12 081.}	91.3809	1.22063	Reff	0.928833	0.5916	{0.260033, 0.925011}	48.1643	ar	90 908.	173 391.	{162 777., 199 446.}	90.7318
27	R0	{2.76831, 19.4155}	{2.47184, 7.68578}	{{0.843585, 5.92184}, {3., 8.89086}}	{22.7611, 60.4142}	inf	25 708.	3811.14	{387.341, 3899.42}	94.299	1.32311	newly	8776.	2519.3	{274., 3705.}	94.4979	1.04271	Reff	0.75287	0.299532	{0.0878732, 0.586091}	62.4993	ar	92 175.	183 809.	{173 287., 194 586.}	99.4133
28	R0	{2.96609, 16.8903}	{2.52549, 5.18743}	{{1.51804, 3.62919}, {3., 11.1337}}	{16.2036, 69.2875}	inf	28 896.	12 392.	{693.215, 42 339.6}	87.0801	0.883913	newly	11 093.	13 920.3	{653., 40 837.}	111.465	0.586033	Reff	0.743925	0.694219	{0.33407, 1.26677}	40.4861	ar	94 024.	205 188.	{172 158., 283 081.}	118.229
29	R0	{2.17046, 14.39}	{2.52178, 12.3898}	{{1.94677, 3.81654}, {3., 18.7685}}	{25.0906, 41.2788}	inf	18 683.	8185.	{514.518, 8496.82}	100.386	0.971476	newly	8778.	6572.9	{147., 25 835.}	111.976	0.950918	Reff	0.946993	0.556422	{0.142143, 1.94672}	68.7158	ar	84 001.	193 088.	{164 843., 271 111.}	129.864
30	R0	{2.88772, 12.4647}	{2.21973, 6.03728}	{{0.794062, 3.19791}, {3., 9.09487}}	{27.5645, 51.565}	inf	28 167.	2756.74	{288.859, 1286.35}	95.3676	1.48782	newly	14 527.	2264.11	{281., 1319.}	97.1034	1.29764	Reff	0.836804	0.355908	{0.111138, 0.506894}	67.8426	ar	93 273.	182 638.	{172 520., 192 154.}	95.8097
31	R0	{2.46831, 8.14308}	{2.15772, 5.06498}	{{0.0469129, 3.92737}, {3., 6.46459}}	{35.042, 38.4789}	inf	22 336.	2701.66	{195.02, 4148.56}	89.5484	1.22727	newly	17 282.	3167.83	{136., 4568.}	91.5375	1.18221	Reff	0.883087	0.307009	{0.0050369, 0.539757}	70.7485	ar	88 762.	169 894.	{161 171., 192 274.}	96.5536
32	R0	{1.93236, 7.38987}	{2.28275, 11.2502}	{{0.192481, 4.39489}, {3., 16.3647}}	{42.225, 69.9523}	inf	13 853.	5190.99	{1203.54, 8837.97}	72.8262	0.621678	newly	12 032.	4922.88	{805., 6476.}	91.5931	0.783766	Reff	0.884615	0.692907	{0.11567, 1.97878}	54.228	ar	77 654.	199 607.	{181 551., 252 371.}	157.046
33	R0	{2.82251, 17.6954}	{2.11862, 6.02867}	{{0.384369, 1.44462}, {3., 17.7287}}	{32.1155, 66.0004}	inf	27 972.	8098.87	{498.311, 7518.21}	79.4863	0.891568	newly	10 722.	10 373.7	{399., 13 039.}	88.3379	0.598856	Reff	1.00304	0.464573	{0.0607252, 0.677462}	60.1016	ar	92 745.	199 921.	{179 223., 246 492.}	115.56
34	R0	{1.87083, 12.3994}	{2.46997, 10.3716}	{{1.63505, 1.42352}, {3., 19.3938}}	{41.61, 69.8711}	inf	12 842.	2496.05	{894.905, 3214.22}	83.8241	0.819501	newly	7135.	3125.43	{463., 7476.}	79.2408	0.68774	Reff	1.01449	0.713407	{0.161842, 1.51213}	51.6371	ar	76 282.	186 813.	{170 386., 235 029.}	144.898
35	R0	{2.95779, 16.3003}	{2.19941, 5.56347}	{{0.358114, 1.337}, {3., 17.8968}}	{27.2954, 70.1662}	inf	29 124.	7479.87	{311.827, 6685.47}	88.6099	1.09852	newly	12 158.	7925.03	{319., 10 486.}	93.5977	0.76574	Reff	1.01659	0.356811	{0.0621839, 0.735753}	64.9013	ar	93 873.	185 564.	{167 019., 234 565.}	97.6759
36	R0	{2.18718, 14.261}	{2.52566, 5.84395}	{{1.71269, 3.25566}, {3., 8.21358}}	{29.7404, 59.0214}	inf	18 672.	3245.86	{965.879, 5284.36}	88.2974	0.996601	newly	8858.	3687.87	{573., 7496.}	86.7928	0.757392	Reff	0.915641	0.437531	{0.125711, 0.657078}	56.3199	ar	84 379.	176 251.	{163 344., 185 874.}	108.88
37	R0	{2.44912, 9.31118}	{2.50471, 8.11272}	{{2.11822, 2.31861}, {3., 11.6987}}	{17.5242, 38.8624}	inf	21 427.	8134.97	{3921.27, 6692.58}	81.026	0.628839	newly	14 344.	8977.14	{2346., 21 946.}	89.4979	0.596222	Reff	0.796601	0.681478	{0.293882, 2.11507}	66.4319	ar	88 442.	200 627.	{179 247., 266 870.}	126.846
38	R0	{2.05949, 5.72514}	{2.5227, 7.04255}	{{0.824847, 2.73028}, {3., 10.6422}}	{36.0564, 57.3643}	inf	16 011.	8788.69	{4893.34, 11 469.5}	61.1731	0.381495	newly	16 782.	9672.62	{3674., 18 725.}	70.2233	0.454147	Reff	0.827297	0.767397	{0.46939, 0.811044}	40.9019	ar	81 587.	201 372.	{184 123., 223 793.}	146.819
39	R0	{1.75505, 8.52404}	{2.1317, 7.50721}	{{0.633811, 1.8015}, {3., 11.2202}}	{59.4052, 43.8223}	inf	10 952.	2746.05	{155.35, 2882.54}	98.9552	1.18182	newly	8590.	2119.01	{129., 4551.}	94.612	1.19422	Reff	0.957748	0.372017	{0.0853418, 0.94902}	63.1553	ar	70 925.	188 484.	{178 370., 205 099.}	165.751
40	R0	{2.24724, 13.7861}	{2.55811, 6.1048}	{{1.67115, 3.39466}, {3., 8.14358}}	{27.6488, 55.7177}	inf	18 516.	3462.38	{784.873, 5346.43}	91.7519	1.05024	newly	8935.	3826.34	{470., 7594.}	91.9693	0.841284	Reff	0.839861	0.363839	{0.0108075, 0.684971}	57.7285	ar	85 257.	174 379.	{162 419., 192 758.}	104.533
41	R0	{1.77635, 8.62406}	{2.72221, 5.77997}	{{2.23351, 3.82015}, {3., 6.26993}}	{53.2472, 32.9785}	inf	11 281.	6282.87	{1614.43, 10 766.8}	81.3371	0.54815	newly	8585.	8467.43	{1197., 19 537.}	111.74	0.540171	Reff	0.87725	0.586021	{0.203197, 1.23328}	59.4715	ar	72 941.	186 941.	{167 262., 209 773.}	156.291
42	R0	{1.80397, 5.4932}	{2.50908, 7.29352}	{{0.847841, 2.48896}, {3., 10.5078}}	{52.1808, 63.173}	inf	10 692.	5281.14	{1834.17, 6874.9}	80.661	0.564269	newly	11 817.	5078.14	{1507., 5822.}	82.7532	0.639709	Reff	0.8332	0.723577	{0.49291, 0.80494}	35.7314	ar	73 232.	197 790.	{183 527., 206 636.}	170.086
43	R0	{2.83096, 16.7077}	{2.71504, 9.68516}	{{2.18582, 4.1345}, {3., 13.23}}	{11.8573, 42.0318}	inf	27 789.	3323.72	{847.242, 3833.86}	89.0868	1.1412	newly	11 209.	3422.23	{505., 2870.}	96.3232	0.957129	Reff	0.932207	0.726935	{0.00884745, 1.82791}	65.0994	ar	92 740.	189 796.	{168 470., 237 465.}	104.654
44	R0	{2.73678, 11.6021}	{2.57436, 10.4437}	{{1.91464, 4.11052}, {3., 14.6658}}	{16.8993, 37.3928}	inf	23 763.	7146.53	{1668.91, 12 486.7}	69.9258	0.636158	newly	12 451.	7394.08	{1193., 8733.}	85.46	0.581166	Reff	0.638655	0.900413	{0.558386, 1.91462}	46.3226	ar	92 003.	208 254.	{183 789., 281 434.}	126.355
45	R0	{2.14235, 6.98601}	{2.67701, 10.2884}	{{1.50863, 3.63555}, {3., 19.8223}}	{35.0569, 86.8006}	inf	15 339.	9601.06	{1735.2, 20 517.7}	79.7321	0.558017	newly	13 010.	9870.81	{983., 25 071.}	106.32	0.694075	Reff	0.718266	0.628346	{0.233565, 1.49778}	54.2567	ar	82 974.	213 553.	{189 164., 292 507.}	157.374
46	R0	{2.23409, 9.21289}	{2.5547, 5.74444}	{{1.54141, 3.57745}, {3., 11.3384}}	{28.6004, 49.6445}	inf	19 256.	8490.59	{1355.06, 13 776.1}	90.6946	0.777479	newly	14 108.	10 798.8	{840., 14 708.}	101.472	0.670277	Reff	0.983357	0.727116	{0.126971, 0.972535}	44.7859	ar	85 163.	193 416.	{169 762., 260 819.}	127.112
47	R0	{2.12258, 8.15265}	{2.55164, 6.38771}	{{2.28211, 2.31735}, {3., 10.4029}}	{32.19, 42.744}	inf	17 054.	7849.74	{2079.21, 8949.25}	91.4122	0.705037	newly	13 477.	9792.96	{1455., 29 187.}	96.4004	0.613096	Reff	0.871829	0.865844	{0.241511, 2.00355}	40.3001	ar	82 690.	210 262.	{186 375., 280 033.}	154.278
48	R0	{2.71771, 10.4636}	{2.55276, 7.58887}	{{0.902719, 2.66527}, {3., 10.9317}}	{17.2875, 31.4907}	inf	26 340.	7378.54	{3770.18, 17 424.5}	73.358	0.652844	newly	16 548.	8276.15	{2667., 20 414.}	78.5068	0.572181	Reff	0.927228	0.753773	{0.255269, 2.16066}	56.3405	ar	91 716.	206 562.	{184 550., 280 091.}	125.22
49	R0	{2.44369, 6.66477}	{2.49144, 4.95817}	{{1.58827, 3.73631}, {3., 6.47088}}	{25.6288, 25.6063}	inf	18 124.	2024.69	{386.083, 2187.2}	88.8287	1.21147	newly	14 957.	2031.42	{313., 2078.}	87.52	1.18385	Reff	0.592717	0.264235	{0.0218448, 0.394264}	60.627	ar	88 827.	177 281.	{172 764., 204 647.}	105.562
50	R0	{2.77383, 17.9775}	{2.63622, 9.29727}	{{2.19979, 3.88615}, {3., 13.59}}	{14.7454, 48.2838}	inf	25 584.	2618.55	{1115.11, 6197.64}	89.7649	1.08267	newly	9271.	2503.24	{632., 9176.}	78.0794	0.815433	Reff	0.747852	0.552686	{0.0793327, 1.27458}	62.0219	ar	92 334.	191 940.	{179 603., 213 430.}	107.876
